# Acetyl-coenzyme A: A metabolic master regulator of autophagy and longevity

**DOI:** 10.4161/auto.28919

**Published:** 2014-05-15

**Authors:** Sabrina Schroeder, Tobias Pendl, Andreas Zimmermann, Tobias Eisenberg, Didac Carmona-Gutierrez, Christoph Ruckenstuhl, Guillermo Mariño, Federico Pietrocola, Alexandra Harger, Christoph Magnes, Frank Sinner, Thomas R Pieber, Jörn Dengjel, Stephan J Sigrist, Guido Kroemer, Frank Madeo

**Affiliations:** 1 Institute of Molecular Biosciences; University of Graz; Graz, Austria; 2 INSERM U848; Pavillon de Recherche 1; Villejuif, France; 3 Metabolomics and Cell Biology Platforms; Institut Gustave Roussy; Pavillon de Recherche 1; Villejuif, France; 4 Université Paris Sud; Faculté de Médecine; Le Kremlin Bicêtre; France; 5 Division of Endocrinology and Metabolism; Dept. of Internal Medicine; Medical University of Graz; Graz, Austria; 6 HEALTH–Institute for Biomedicine and Health Sciences; Joanneum Research Forschungsgesellschaft m.b.H.; Graz, Austria; 7 Department of Dermatology; University Freiburg Medical Center; Freiburg, Germany; 8 Institute for Biology/Genetics; Freie Universität; Berlin, Germany; 9 NeuroCure; Charité; Berlin, Germany; 10 Equipe 11 labellisée Ligue contre le cancer; INSERM U1138; Centre de Recherche des Cordeliers; Paris, France; 11 Pôle de Biologie; Hôpital Européen Georges Pompidou; AP-HP; Paris, France; 12 Université Paris Descartes; Sorbonne Paris Cité; Paris, France

**Keywords:** autophagy, aging, acetyl-coenzyme A, histone acetylation, transcription, epigenetics, ATG

## Abstract

As the major lysosomal degradation pathway, autophagy represents the guardian of cellular homeostasis, removing damaged and potentially harmful material and replenishing energy reserves in conditions of starvation. Given its vast physiological importance, autophagy is crucially involved in the process of aging and associated pathologies. Although the regulation of autophagy strongly depends on nutrient availability, specific metabolites that modulate autophagic responses are poorly described. Recently, we revealed nucleo-cytosolic acetyl-coenzyme A (AcCoA) as a phylogenetically conserved inhibitor of starvation-induced and age-associated autophagy. AcCoA is the sole acetyl-group donor for protein acetylation, explaining why pharmacological or genetic manipulations that modify the concentrations of nucleo-cytosolic AcCoA directly affect the levels of protein acetylation. The acetylation of histones and cytosolic proteins inversely correlates with the rate of autophagy in yeast and mammalian cells, respectively, despite the fact that the routes of de novo AcCoA synthesis differ across phyla. Thus, we propose nucleo-cytosolic AcCoA to act as a conserved metabolic rheostat, linking the cellular metabolic state to the regulation of autophagy via effects on protein acetylation.

Autophagy is regulated by nutrient- and energy-sensing pathways and the subsequent activity of protein kinases, such as the well-known aging regulators MTOR (mechanistic target of rapamycin; Tor in yeast), AMPK (AMP-dependent protein kinase; Snf1 in yeast) and AKT/protein kinase B (Sch9 in yeast). However, recent studies suggest that apart from phosphorylation, other posttranslational modifications, in particular acetylation of proteins, may also influence autophagy by targeting pathways that are, at least partly, different from that of known kinase regulators. Multiple regulators and core components of the autophagic machinery undergo changes in their acetylation status, and acetylation of histone lysine residues has been linked to different aspects of autophagy.

Besides peroxisomal β-oxidation that creates AcCoA by the breakdown of fatty acids, in the yeast *Saccharomyces cerevisiae* AcCoA is synthesized by a pathway that fuels 2 distinct, separate intracellular pools: the mitochondrial vs. the nucleo-cytosolic pool. The putative CoA transferase Ach1 and the mitochondrial AcCoA synthetase isoform Acs1 generate AcCoA in mitochondria from acetate, while the pyruvate dehydrogenase complex converts pyruvate into mitochondrial AcCoA by oxidative decarboxylation. The predominantly nuclear-localized yeast AcCoA synthetase, Acs2, catalyzes the formation of AcCoA from acetate and CoA within the nucleo-cytosolic compartment. Acs2 is essential for histone acetylation, when cells grow on glucose as a carbon source. Similarly, in mammals AcCoA is produced in mitochondria from pyruvate, fatty acids or branched-chain amino acids. In contrast to yeast, the nucleo-cytosolic AcCoA production in mammals is mostly executed by ACLY (ATP citrate lyase), which ultimately converts citrate exported from mitochondria into AcCoA. However, it should be emphasized that in mammals, a cytosolic AcCoA synthetase contributes to the nucleo-cytosolic pool of AcCoA. Although the pathways through which AcCoA is synthesized are largely species-dependent, we could demonstrate that the nucleo-cytosolic pool was more important for repressing autophagy than the mitochondrial pool, both in yeast and in mammals (Fig. 1).

**Figure:**
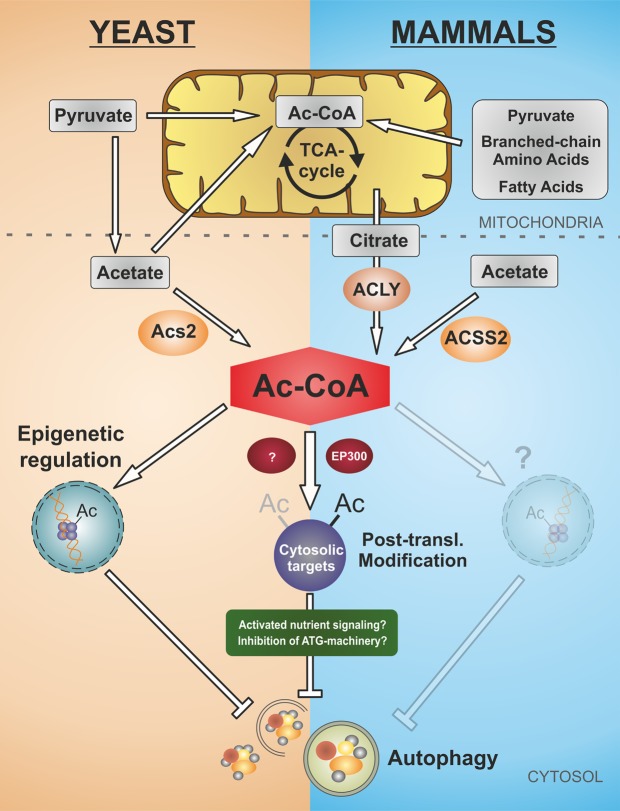
**Figure 1.** Nucleo-cytosolic AcCoA determines the autophagic response in yeast and mammals. In *Saccharomyces cerevisiae* grown on glucose, the major source for nucleo-cytosolic AcCoA is the AcCoA synthetase 2 (Acs2), which uses acetate as a substrate. Mitochondria solely influence cytosolic AcCoA levels by removing acetate and its precursor pyruvate, fueling the tricarboxylic acid (TCA) cycle. In contrast, cytosolic AcCoA production in mammals requires a detour via mitochondria, since it depends on citrate derived from the TCA cycle, which is fed by pyruvate as well as by fatty acids and branched-chain amino acids. Citrate is exported from mitochondria and converted to AcCoA by the cytosolic ATP citrate lyase (ACLY). In both models, high concentrations of nucleo-cytosolic AcCoA favor protein acetylation by acetyltransferases. Protein hyperacetylation subsequently inhibits autophagy by epigenetic regulation of autophagy-related genes, by direct posttranslational inactivation of proteins engaged in the autophagic machinery, as well as by (direct or indirect?) modulation of nutrient-sensing kinase pathways.

We found that nucleo-cytosolic AcCoA production limited autophagy in aging yeast, meaning that even a partial knockdown of the *ACS2* gene sufficed to enhance autophagy upon chronological aging. In contrast, blockage of the mitochondrial route of AcCoA formation by deletion of the CoA-transferase *ACH1* or the mitochondrial pyruvate carrier *MPC1* caused an age-dependent defect in autophagic flux, as it hyperactivated the nucleo-cytosolic Acs2 pathway, culminating in histone hyperacetylation and transcriptional repression of the autophagy essential gene *ATG7.* Corroborating this idea, simultaneous knockdown of *ACS2* in the *ACH1* deletion background reinstated the expression of *ATG7*, as it abolished the autophagy defect and the accelerated aging phenotype. This led us to conclude that the nucleo-cytosolic rather than the mitochondrial AcCoA pool suppresses autophagy. Interestingly, inhibition of nutrient-signaling, for example by additional deletion of *SCH9* or Tor complex 1 inhibition by rapamycin, failed to abolish the detrimental effects of increased nucleo-cytosolic AcCoA production on autophagy.

We also assessed the capacity of AcCoA to regulate starvation-induced autophagy in cell culture and mice by pharmacologically or genetically manipulating major routes of mammalian AcCoA formation, such as the pyruvate dehydrogenase complex or ACLY. Reduced or increased cytosolic AcCoA directly affected protein acetylation as it induced or suppressed autophagy, respectively. In line with the data obtained from yeast, changes in the nucleo-cytosolic fraction of AcCoA were finally causal for the observed impact on autophagy, since inactivation of purely cytosolic enzymes involved in AcCoA generation (e.g., ACLY or the mammalian AcCoA synthetase ACSS2) was sufficient to stimulate autophagy and, even more importantly, blockage of ACLY prevented autophagy suppression induced by mitochondrial AcCoA overproduction.

Of note, while autophagy regulation by AcCoA during aging seems to be independent of well-known kinase signaling pathways (such as those involving Tor and Sch9) in yeast, acute nutrient depletion was accompanied by dephosphorylation of MTOR substrates and the activation of AMPK in mammalian cells. Furthermore, high rates of AcCoA counteracted autophagy induction by rapamycin or AKT inhibitors in mammals. It is therefore tempting to speculate that, at least in a short-term context, nucleo-cytosolic AcCoA depletion might activate energy-sensing kinase pathways via yet-to-be-explored mechanisms. Nevertheless, high AcCoA concentrations might also block the core autophagy machinery (e.g., by acetylation of *ATG* gene products) downstream of nutrient kinase targets. It remains to be explored to which extent (and on which time scale) cytoplasmic and epigenetic alterations explain the relationship between AcCoA accumulation, protein hyperacetylation, and autophagy repression.

Our results clearly demonstrate the central importance of the nucleo-cytosolic AcCoA pool for autophagy regulation and suggest the possibility of inhibiting or stimulating autophagic flux by targeting AcCoA metabolism.

## Notes

EisenbergTSchroederSAndryushkovaAPendlTKüttnerVBhukelAMariñoGPietrocolaFHargerAZimmermannA Nucleocytosolic depletion of the energy metabolite acetyl-coenzyme a stimulates autophagy and prolongs lifespan Cell Metab 2014 19 431 44 10.1016/j.cmet.2014.02.010

MariñoGPietrocolaFEisenbergTKongYMalikSAAndryushkovaASchroederSPendlTHargerANiso-SantanoM Regulation of autophagy by cytosolic acetyl-coenzyme A Mol Cell 2014 53 710 25 10.1016/j.molcel.2014.01.016

